# Nutritional Changes and Contributing Factors in Iran: A Comprehensive Study From PERSIAN Cohort Study (2015–2017 and 2021–2023)

**DOI:** 10.1002/fsn3.72060

**Published:** 2026-06-25

**Authors:** Farid Najafi, Neda Izadi, Shahab Rezaeian, Yahya Pasdar, Fatemeh Khosravi Shadmani, Amir Bagheri, Hossein Poustchi, Sareh Eghtesad, Farhad Pourfarzi, Azim Nejatizadeh, Mojtaba Farjam, Farahnaz Joukar, Amir Hoshang Bavarsad, Mohammad Reza Fattahi, Motahareh Kheradmand, Ali Ahmadi, Mohammad Hossein Somi, Iraj Mohebbi, Sayed Bahman Panahande, Hassan Mozaffari‐Khosravi, Alireza Ansari‐Moghadam, Masoumeh Ghoddusi Johari, Habib Allah Shahryari

**Affiliations:** ^1^ Research Center for Environmental Determinants of Health (RCEDH), Health Institute Kermanshah University of Medical Sciences Kermanshah Iran; ^2^ Research Center for Social Determinants of Health, Research Institute for Endocrine Sciences Shahid Beheshti University of Medical Sciences Tehran Iran; ^3^ Infectious Diseases Research Center, Health Institute Kermanshah University of Medical Sciences Kermanshah Iran; ^4^ Social Determinants of Health Research Center, Social Health Research Institute University of Social Welfare and Rehabilitation Sciences Tehran Iran; ^5^ Department of Social Welfare Management University of Social Welfare and Rehabilitation Sciences Tehran Iran; ^6^ Department of Nutritional Sciences, School of Nutritional Sciences and Food Technology Kermanshah University of Medical Sciences Kermanshah Iran; ^7^ Liver and Pancreatobiliary Diseases Research Center, Digestive Disease Research Institute Tehran University of Medical Sciences Tehran Iran; ^8^ Digestive Disease Research Center Ardabil University of Medical Sciences Ardabil Iran; ^9^ Research Center for Molecular Medicine Hormozgan University of Medical Sciences Bandar Abbas Iran; ^10^ Noncommunicable Diseases Research Center Fasa University of Medical Sciences Fasa Iran; ^11^ Gastrointestinal and Liver Diseases Research Center Guilan University of Medical Sciences Rasht Iran; ^12^ Gastroentrology and Hepatology Center, School of Medicine Ahvaz Jondishapour University of Medical Sciences Ahvaz Iran; ^13^ Gastroenterohepatology Research Center Shiraz University of Medical Sciences Shiraz Iran; ^14^ Gastrointestinal Cancer Research Center, Non‐Communicable Diseases Institute Mazandaran University of Medical Sciences Sari Iran; ^15^ Health Sciences Research Center, Addiction Institute Mazandaran University of Medical Sciences Sari Iran; ^16^ Modeling in Health Research Center Shahrekord University of Medical Sciences Shahrekord Iran; ^17^ Liver and Gastrointestinal Diseases Research Center Tabriz University of Medical Sciences Tabriz Iran; ^18^ Social Determinants of Health Research Center Urmia University of Medical Sciences Urmia Iran; ^19^ Department of Nutrition, School of Health and Nutrition Yasuj University of Medical Sciences Yasuj Iran; ^20^ Department of Nutrition, School of Public Health Shahid Sadoughi University of Medical Sciences Yazd Iran; ^21^ Health Promotion Research Center Zahedan University of Medical Sciences Zahedan Iran; ^22^ Breast Diseases Research Center Shiraz University of Medical Sciences Shiraz Iran; ^23^ School of Public Health Kermanshah University of Medical Sciences Kermanshah Iran

**Keywords:** dietary change, generalized estimating equations, macronutrient, PERSIAN cohort, socioeconomic status

## Abstract

In recent decades, notable changes have occurred in dietary patterns across different societies. Using PERSIAN cohort data, we investigated dietary changes among Iranian people in two stages: baseline phase (2015–2017) and reassessment phase (2021–2023). Additionally, the factors influencing these changes will also be examined. The PERSIAN cohort is a multicenter and prospective study comprising 32,000 individuals aged 35–70 years from 18 geographical regions of Iran. The study's data were collected using a 113‐item Food Frequency Questionnaire assessing dietary intake over the past year. The generalized estimating equations (GEE) method was employed to analyze changes in macronutrients and total energy across two time periods. The effects of age, time, gender, place of residence, marital status, education, socioeconomic status (SES), smoking, physical activity, body mass index, DMF (decayed, missing, and filled teeth), sleep duration, hypertension, diabetes, cardiovascular disease, metabolic syndrome, and center were also assessed in the study. Total energy intake in the reassessment phase (2021–2023) was reduced by 250 kcal/day compared with the baseline phase (2015–2017), accompanied by reductions in daily carbohydrate (40.3 g/day), protein (10.3 g/day), and fat (5.7 g/day) intake. In adjusted analyses, women consumed less carbohydrate, protein, fat, and total energy than men: 82.91 g/day, 17.36 g/day, 9.15 g/day, and 485.34 kcal/day, respectively. In addition, there was a lower decline in the intake of all macronutrients in women compared to men. Rural dwellers had significantly higher daily intakes of carbohydrates (11.17 g/day), protein (1.37 g/day), fat (1.57 g/day), and total energy (96.21 kcal/day). Individuals with obesity had a significantly higher average intake of protein (4.82 g/day), fat (3.64 g/day), and total energy (195.62 kcal/day). In the multivariable analysis, individuals in the highest SES group consumed significantly more protein (2.28 g/day), total fat (3.39 g/day), and total energy (47.15 kcal/day) compared to those in the lowest SES group. In addition, all SES received lower intake of macronutrients over time homogenously. Patients with hypertension, diabetes, and cardiovascular disease consumed significantly lower average total energy (31.62, 67.61, and 79.35 kcal/day, respectively) compared to healthy individuals; conversely, those with metabolic syndrome consumed 17.12 kcal/day more energy than their healthy counterparts. Economic challenges and social inequalities have significantly impacted the intake of macronutrients in the Iranian diet, highlighting the need for targeted nutritional interventions to improve dietary balance, particularly among vulnerable populations. Food subsidies for lower‐income deciles could be a beneficial intervention.

## Graphical abstract

From baseline to reassessment phase, Iranians experienced significant reductions in energy and macronutrient intakes. Sociodemographic characteristics, lifestyle, anthropometric measures, and health status are important contributors to these changes, highlighting the impact of economic pressure and nutritional inequality in Iran.

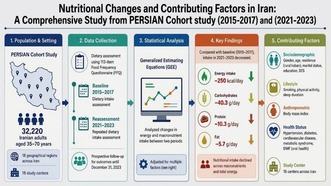

## 1 Introduction

Over the past decades, significant changes have occurred in the dietary patterns of people around the world, influenced by economic, social, and environmental factors (Béné et al. 2019). These changes particularly include a transition from simple, minimally processed foods to diets that are rich in meat, vegetable oils, and processed foods such as fast foods, packaged snacks, and sugar‐sweetened beverages (Whitney et al. 2019). This trend is associated with a rise in obesity and chronic diseases (Baker and Friel 2014). Although these dietary changes are mainly observed in industrialized countries, nonindustrialized countries have a similar pattern (Islam et al. 2021). Many Middle Eastern countries have experienced a rise in the prevalence of obesity due to a shift toward a Western lifestyle (Okati‐Aliabad et al. 2022). This lifestyle change increases the risk of obesity, metabolic syndrome, diabetes, and hypertension in these countries (Mirzaei et al. 2017; Pasdar et al. 2015). Numerous studies have emphasized a significant relationship between dietary changes and the rising prevalence of several noncommunicable diseases, including obesity, diabetes, cardiovascular diseases, and certain types of cancer (Popkin 2006; Yang et al. 2012; Zhang et al. 2021; Hu et al. 2015).

All foods contain three types of macronutrients: carbohydrates, fats, and proteins. One common method for analyzing foods and their changes is to evaluate the amounts of these macronutrients, which can have a significant impact on our health (Shan et al. 2019). Macronutrients are not only the primary source of energy for the body but also play a vital role in maintaining health (Carreiro et al. 2016; Muth and Park 2021). Therefore, changes in the consumption of these nutrients at the population level, especially during periods of economic development and urbanization, can significantly impact individual health (Seidelmann et al. 2018; Alhazmi et al. 2012).

Monitoring the nutritional status of the population is essential for the development and evaluation of health policies. Many countries monitor their food consumption through population‐based national surveys (Saito et al. 2018; Gose et al. 2016; Dinnissen et al. 2021; Abufaraj et al. 2021). In Iran, several studies, including the Tehran Lipid and Glucose Cohort Study (Azizi et al. 2009), Golestan Cohort Study (Poustchi et al. 2014), and the Isfahan Heart Study (Sarrafzadegan et al. 2011), have been conducted as single‐center studies. These studies have performed limited examinations of dietary changes in their covered populations. In contrast, the Prospective Epidemiological Research Studies in Iran (PERSIAN Cohort) was launched in 2014, encompasses participants from all ethnicities and cultures across Iran (Zare Sakhvidi et al. 2021). The reassessment phase of this cohort study was conducted in 2021, and the information of the same individuals was re‐examined in the baseline phase. It is noteworthy that the years during which the PERSIAN Cohort was conducted coincided with significant economic challenges faced by the Iranian population. For instance, the inflation rate in various consumer items (such as meat at 116%, bread and grains at 58%, oils and fats at 240%, milk, cheese, and eggs at 83.5%, and vegetables and legumes at 68.5%) has risen sharply (Hejazi and Emamgholipour 2020; Iran SCo 2023). Other economic factors such as unemployment, the growth of marginalization, poverty, and the lack of suitable job opportunities have also increased (Zeinalabedini et al. 2023). Consequently, society has faced numerous challenges over the years that could have profoundly affected nutritional status and health. These challenges include issues such as overweight, obesity, hypertension, diabetes, cardiovascular diseases, osteoporosis, anemia, and various types of cancers (Hejazi and Emamgholipour 2020).

Therefore, this research aims to examine the changes in macronutrient intake among the people of Iran. We will use data from the PERSIAN Cohort across two phases: the baseline phase (in 2015) and the reassessment phase (in 2021). Additionally, we will identify the factors influencing these changes, such as age, time, gender, place of residence, education, body mass index, socioeconomic status, physical activity, hypertension, diabetes, etc. This research is particularly important given the current social and economic conditions, where numerous challenges have impacted the nutritional status and health of the community.

## 2 Materials and Methods

### 2.1 Study Design and Population

**FIGURE 1:**
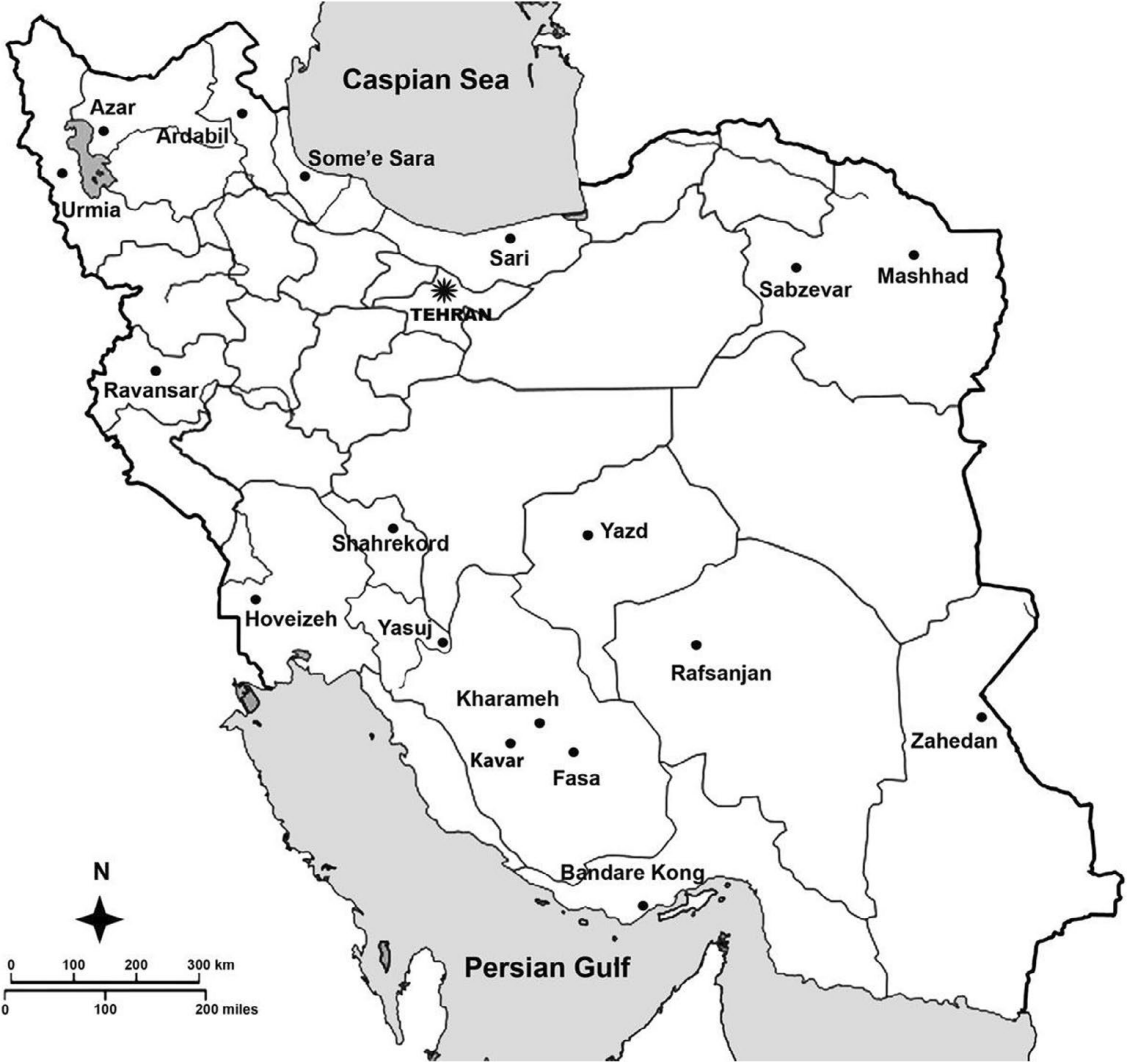
Locations of research centers in the PERSIAN Cohort Study, Iran, 2014–2021. The sites were chosen so that all of the major ethnic groups in Iran, as well as variable climates and geographical regions, are included. PERSIAN, Prospective Epidemiological Research Studies in Iran.

The data for this study comes from the enrolment and reassessment phases of the PERSIAN cohort, a multicenter, prospective cohort study investigating the roles of metabolic, dietary, lifestyle, and environmental factors in the development of chronic diseases (Poustchi et al. 2018). The cohort includes a sample size of 180,000 individuals aged 35–70 years from 18 geographically distinct areas of Iran. While the Ministry of Health and Medical Education oversees the project, investigators at universities in each province conduct the research. These sites were chosen to assess a wide range of cultural, genetic, lifestyle, and environmental exposures that may positively or negatively impact disease development, encompassing all ethnicities as well as different geographical areas of Iran, both urban and rural. The locations of the PERSIAN cohort sites are shown in Figure 1, with more details provided elsewhere (Poustchi et al. 2018).

**FIGURE 2:**
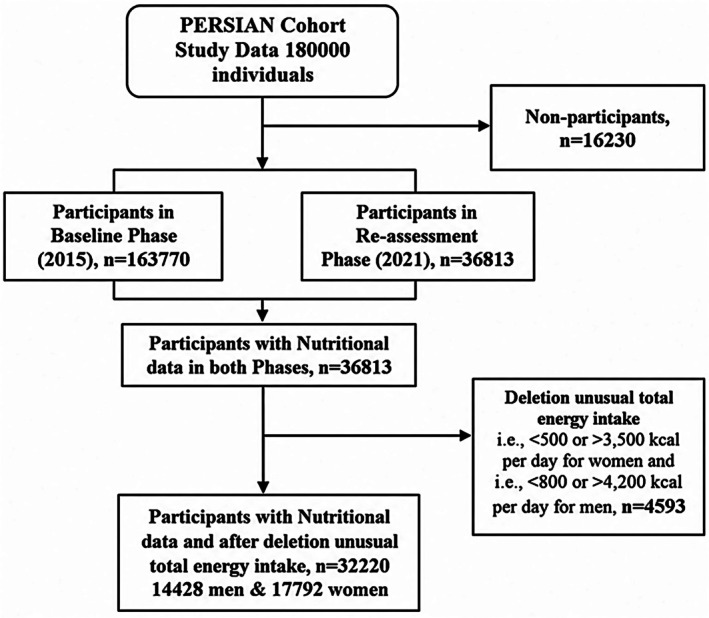
Flowchart of the study participants.

Out of 180,000 individuals, 163,770 participated in the study. This study was conducted in accordance with the guidelines set forth in the Declaration of Helsinki, and all procedures related to study participants were approved by the Ethics Committee of the Ministry of Health, Treatment, and Medical Education, the Research Institute of Gastroenterology and Liver Diseases, Tehran University of Medical Sciences, Iran (Poustchi et al. 2018). This cohort study was approved by the Ethics Committee of Kermanshah University of Medical Sciences (No: KUMS.REC.1403.095). Following the completion of the pilot phase, the baseline phase commenced in March 2015. Baseline data were collected from March 2015 to December 2017. Baseline data were systematically gathered over a recruitment period spanning 24–30 months across all 18 designated study sites. Trained personnel conducted visits to households to verify eligibility and individuals who consented to participate were then scheduled to attend their local cohort center for formal enrollment and initial assessments (Poustchi et al. 2018). Then, for the reassessment phase conducted 6 years later (2021), due to financial and logistical constraints, only 30% of the baseline sample were followed up. This subsample was randomly selected to ensure it remained representative of the original cohort. Therefore, a total of 36,813 individuals was reassessed for their medical, demographic, behavioral, and nutritional statuses. In accordance with the study's objectives, individuals with unusual energy consumption (< 500 or > 3500 kcal/day for women, and < 800 or > 4200 kcal/day for men) were excluded (Heidari et al. 2019; Teymoori et al. 2017). Finally, a total of 32,220 individuals were selected from the 36,813 participants who had repeated assessments of their nutritional status. These participants were drawn from all 18 study sites, ensuring that the subsample remained representative of the original cohort (Figure 2).

### 2.2 Measurements and Definitions

Demographic characteristics and additional information were collected face‐to‐face using a digital questionnaire. Socioeconomic status (SES) was assessed using the Wealth Score Index (WSI), a composite measure based on household assets, housing characteristics, and access to welfare facilities within the PERSIAN cohort. The index was derived using principal component analysis (PCA), and participants were categorized into five socioeconomic groups ranging from the poorest to the richest according to standardized PERSIAN cohort protocols (Poustchi et al. 2018; Moftakhar et al. 2023; Cheraghian et al. 2020).

An interviewer‐administered questionnaire was used to assess smoking status. Individuals were categorized into three groups based on their smoking habits: current smokers, former smokers, and never smokers. Current smokers were defined as those who smoked at least one cigarette daily and had consumed over 100 cigarettes in their lifetime. Never smokers were individuals who had never smoked or had smoked fewer than 100 cigarettes in their lifetime. Former smokers were those who did not smoke regularly or occasionally in the past year but had smoked more than 100 cigarettes in their lifetime (Rezaei et al. 2017). Physical activity was assessed using a validated questionnaire derived from the PERSIAN Cohort Study. Participants reported the duration of various activities—including occupational, household, and recreational—over a 24‐h period. These activities were converted into metabolic equivalents (METs), where 1 MET represents the energy expenditure at rest (3.5 mL O_2_/kg body weight/min). Total daily METs were calculated by summing the MET values of all reported activities and were used as a continuous covariate in the statistical analyses (Poustchi et al. 2018; Kazemi Karyani et al. 2019). Body mass index (BMI) was used to assess weight relative to height. Based on the calculated BMI values, subjects were categorized as follows: those with a BMI < 18.5 were classified as underweight, those with 18.5 ≤ BMI < 24.9 as normal weight, those with 25.0 ≤ BMI < 29.9 as overweight, and those with a BMI ≥ 30 as obese. The Decayed, Missing, and Filled (DMF) index was employed as a measure of oral health in this study, calculated as the total number of teeth that were decayed (D), missing (M), and filled (F). Sleep duration refers to the amount of time participants were asked how many hours they sleep during a normal day (Najafi et al. 2022). In this study, metabolic syndrome (MetS) was defined as meeting at least three criteria specified in the Joint Interim Statement (JIS) for Iranian adults. These criteria include: a fasting blood glucose (FBG) level of 100 mg/dL or higher, or the use of blood glucose‐lowering medications; serum triglycerides (TG) of 150 mg/dL or higher, or the use of TG‐lowering medications; high‐density lipoprotein cholesterol (HDL‐C) levels of 40 mg/dL or lower in men and 50 mg/dL or lower in women, or the use of HDL‐C boosting medications; systolic blood pressure (SBP) of 130 mmHg or higher, or diastolic blood pressure (DBP) of 85 mmHg or higher, or the use of blood pressure‐lowering medications; and a waist circumference (WC) of 94 cm or more in men and 80 cm or more in women (Tabatabaei‐Malazy et al. 2021). According to the PERSIAN cohort study protocol, participants with cardiovascular diseases (CVDs) are those with a history of hospitalization and/or treatment for one or more heart diseases, such as stroke, myocardial infarction (MI), and coronary artery disease, and/or those taking medications for CVDs. Diagnosis of type 2 diabetes (T2D) includes FBS levels equal to or greater than 126 mg/dL and/or treatment with hypoglycemic drugs. In addition, subjects with SBP equal to or greater than 140 mmHg and DBP equal to or greater than 90 mmHg, and/or those treated with blood pressure‐lowering medications were classified as having hypertension (HTN) (Pasdar et al. 2019). The variable “time” was categorized into two categories: the first category corresponded to the baseline phase of the study (2015–2017), and the second category corresponded to the reassessment phase (2021–2023).

### 2.3 Food Intake

A validated food frequency questionnaire (FFQ) was used to determine the food intake of the participants in the baseline and reassessment phase (changes of food intake). The FFQ consisted of questions about the frequency of consumption of 113 food items and standard serving sizes (e.g., glass, cup, slice, teaspoon, tablespoon, spatula, and cube). Participants reported the average frequency and portion size of foods consumed in the past year. To minimize recall bias, the FFQ was administered by trained interviewers, and participants were given sufficient time to recall the consumption of each food (Pasdar et al. 2019; Eghtesad et al. 2023, 2024). Local foods were also included in the questionnaire. The amount of each food consumed was converted into grams per day (g/day) using Nutrition IV software to measure the daily intake of food. The validity and reproducibility of the FFQ were further assessed by comparing data from two FFQs completed 1 year apart with the average of twenty‐four 24‐h dietary recalls (24 h recalls) (Eghtesad et al. 2023; Eghtesad et al. 2024). The results showed that the FFQ was suitable for identification of major dietary patterns.

Daily intakes of total energy, carbohydrate, protein, and fat were computed for both phases. These macronutrients were further classified into the following subgroups based on food sources, nutritional quality, and chemical composition.

Carbohydrates were categorized into high‐quality and low‐quality carbohydrates according to food source and degree of processing. High‐quality carbohydrates were defined as those primarily derived from minimally processed sources, including whole grains, fruits, vegetables, and legumes. In contrast, low‐quality carbohydrates were mainly from refined grains, added sugars, and highly processed carbohydrate‐rich foods (Ardisson Korat et al. 2025). Protein intake was divided into plant‐based and animal‐based protein; plant‐based protein was calculated from the contributions of grains, legumes, nuts, seeds, and vegetables, while animal‐based protein was derived from meat, poultry, fish, dairy products, and eggs (Lotfi et al. 2022). Fat intake was categorized according to fatty acid composition into saturated fatty acids (SFA), monounsaturated fatty acids (MUFA), and polyunsaturated fatty acids (PUFA), with subgroup intakes estimated separately using detailed fatty acid composition data for each food item (Petersen et al. 2024).

### 2.4 Statistical Analysis

Quantitative variables were described as mean ± standard deviation (SD) and the qualitative variables as frequency (%). Missing data were minimal (3.35%); therefore, complete‐case analysis was used, and observations with missing values were excluded from the relevant analyses. Changes in the variables between the two phases were analyzed using the paired *t*‐test. To assess the effect of outliers in macronutrient values, paired *t*‐test was conducted both with and without outliers. The inclusion of outliers did not affect the significance of the tests, standard deviation, or means. Therefore, we decided to retain the outliers in the study. Also, changes in mean macronutrient consumption were calculated separately for men and women, reported as mean ± standard deviation.

In this study, the generalized estimating equations (GEE) method was performed for data analysis. This method was chosen for its ability to manage intragroup dependencies and handle repeated or clustered data. GEE provides accurate estimates of model parameters while correcting the standard errors. To estimate the effects of factors influencing changes in mean macronutrients (total energy, carbohydrates, total protein, and total fat), a Gaussian distribution with an identity link function was used. Given that only two measurement periods were available, an unstructured correlation type was utilized (Twisk 2013). The GEE approach was selected to obtain population‐averaged estimates of changes in macronutrient intake over time, while accounting for potential confounding by sociodemographic, lifestyle, anthropometric, and clinical variables (Koppes et al. 2008).

Initially, univariate analyses were conducted between the dependent variables and each independent variable (Table S1). After completing the univariate analyses, multivariable GEE models were examined. Variables with a *p* value below 0.2 in the univariate analyses were included as covariates in the multivariable models. Thus, the final GEE models for each macronutrient and total energy were adjusted for age, time, gender, residence, education status, marital status, socioeconomic status, smoking status, physical activity, BMI categories, DMF index, sleep duration, metabolic syndrome, hypertension, diabetes, and cardiovascular disease as potential confounders. Finally, parameter estimates (beta coefficient) were reported along with the 95% confidence interval (CI). Data were analyzed using STATA software version 17 (Stata Corp, College Station, TX, USA).

## 3 Results

The total sample consisted of 32,220 individuals, including 14,428 men and 17,792 women. The mean age of participants in the baseline phase was 47.5 years (SD = 7.90), with most living in urban areas (71.84%). In terms of education level, the majority of individuals (70.02%) had education below a diploma, and 18.83% of the participants were illiterate. Regarding BMI, the baseline data indicated that 26.76% of participants were classified as normal weight, 71.55% as overweight and obese. From total, 79.45% were nonsmokers (79.45%).

Additionally, participants reported an average sleep duration of 7.11 h (SD = 1.49). Marital status showed that a significant majority were married (93.31%), with a small percentage being single (1.57%) or divorced (0.89%). The analysis also indicated that 38.53% of participants had metabolic syndrome, while 26.63% had a history of CVD. Hypertension was present in 26.47% of the participants, and 16.80% reported having diabetes (Table 1).

**TABLE 1:** Demographic characteristics of the study participants in the PERSIAN cohort study at baseline phases.

	Baseline, *n* (%)		
	Total, *n* = 32,220	Men, *n* = 14,428	Women, *n* = 17,792
Age^a^	47.5 (7.9)	47.6 (7.95)	47.49 (8.01)
Residence type^b^			
Urban	23,148 (71.84)	10,354 (71.76)	12,794 (71.91)
Rural	9072 (28.16)	4074 (28.24)	4998 (28.09)
Education			
Illiterate	6068 (18.83)	1731 (12.00)	4337 (24.38)
Up to Diploma	22,561 (70.02)	10,341 (71.67)	12,220 (68.68)
Up to Bachelor's	3073 (9.54)	1962 (13.60)	1111 (6.24)
Master's Degree and above	518 (1.61)	394 (2.73)	124 (0.70)
Body mass index			
Underweight	543 (1.69)	407 (2.82)	136 (0.76)
Normal	8621 (26.76)	5165 (35.80)	3456 (19.42)
Overweight	13,337 (41.39)	6310 (43.73)	7027 (39.50)
Obesity	9719 (30.16)	2546 (17.65)	7173 (40.32)
Socioeconomic status			
The poorest	5928 (18.43)	1964 (13.64)	3964 (22.31)
Poor	6924 (21.53)	2973 (20.65)	3951 (22.24)
Middle	7020 (21.83)	3251 (22.58)	3769 (21.21)
Rich	7203 (22.39)	3324 (23.09)	3879 (21.83)
The richest	5089 (15.82)	2885 (20.04)	2204 (12.41)
Smoking status			
Non‐smoker	25,579 (79.45)	8179 (56.74)	17,397 (97.87)
Current smoker	2576 (8.05)	2376 (16.48)	200 (1.13)
Former smoker	4039 (12.55)	3860 (26.78)	179 (1.01)
Sleep duration	7.11 (1.49)	6.96 (1.43)	7.23 (1.53)
Marital status			
Single	506 (1.57)	113 (0.78)	393 (2.21)
Married	30,366 (93.31)	14,244 (98.72)	15,822 (88.93)
Widowed	1347 (4.18)	33 (0.23)	1314 (7.39)
Divorced	286 (0.89)	37 (0.26)	249 (1.40)
Other	15 (0.05)	1 (0.01)	14 (0.8)
MET‐final	40.15 (7.46)	41.54 (9.60)	39.02 (4.80)
DMF	17.13 (9.63)	17.86 (10.07)	16.54 (9.21)
Metabolic syndrome			
Yes	12,334 (38.53)	5064 (35.36)	7270 (41.11)
No	19,674 (61.47)	9259 (64.64)	10,415 (58.89)
Hypertension			
Yes	8528 (26.47)	3519 (24.39)	5009 (28.15)
No	23,692 (73.53)	10,909 (75.61)	12,783 (71.85)
Diabetes			
Yes	5412 (16.80)	2152 (14.92)	3260 (18.32)
No	2688 (83.20)	1276 (85.08)	14,532 (81.68)
CVD			
Yes	8581 (26.63)	3145 (21.80)	5436 (30.55)
No	23,639 (73.37)	11,283 (78.20)	12,356 (69.45)

Abbreviation: DMF, decayed, missing, and filled teeth.

^a^ Mean (standard deviation).

^b^ Population count (percentage).

### 3.1 Changes in the Intake of Macronutrients

A significant change was observed in macronutrient intake patterns across the overall population and between genders from 2015 to 2021. Overall, the average total energy intake decreased by 250.1 ± 700.7 kcal/day, with a reduction of 312.9 ± 773.2 kcal/day for men and 199.2 ± 631.3 kcal/day for women. It is worth noting that both genders experienced a decrease in total carbohydrate and protein intake, although the reduction in protein intake was greater in men (12.6 ± 28.4 g/day) than in women (7.9 ± 22.9 g/day). However, the energy derived from different macronutrients did not significantly change. Notably, the population was highly heterogeneous, which is reflected in the large standard deviations observed in some strata (Table 2).

**TABLE 2:** Changes in the intake of macronutrients by gender.

Macronutrient	Total, *n* = 32,220			Men, *n* = 14,428			Women, *n* = 17,792		
	Baseline (2015)	Reassessment (2021)	Difference (2021–2015)	Baseline (2015)	Reassessment (2021)	Difference (2021–2015)	Baseline (2015)	Reassessment (2021)	Difference (2021–2015)
	Mean ± SD^a^								
Total energy (kcal/day)^b^	2369.5 ± 680.4	2127.6 ± 667.6	−250.1 ± 700.7	2669.2 ± 684.5	2356.2 ± 691.5	−312.9 ± 773.2	2141.4 ± 577.7	1942.2 ± 585.2	−199.2 ± 631.3
Total carbohydrate (g/day)	386.7 ± 118.1	346.3 ± 115.7	−40.3 ± 121.7	436.2 ± 119.9	385.5 ± 120.2	−50.6 ± 135.3	346.6 ± 100.1	314.6 ± 101.3	−32.00 ± 108.7
H Q‐carbohydrates (g/day)^c^	142.3 ± 79.5	126.9 ± 73.5	−14.3 ± 77.0	155.4 ± 87.9	137.1 ± 80.2	−18.2 ± 85.4	131.6 ± 70.1	120.5 ± 66.8	−11.1 ± 69.3
L Q‐carbohydrates (g/day)^c^	226.7 ± 109.1	204.4 ± 103.4	−22.3 ± 95.8	261.8 ± 116.1	233.3 ± 110.9	−28.5 ± 107.2	198.2 ± 93.8	180.9 ± 90.4	−17.2 ± 85.1
Total protein (g/day)	78.7 ± 25.0	68.7 ± 23.7	−10.03 ± 25.6	89.13 ± 25.7	76.4 ± 24.9	−12.6 ± 28.4	70.3 ± 21	62.4 ± 20.7	−7.9 ± 22.9
Animal protein (g/day)	29.8 ± 14.6	24.6 ± 13.0	−5.1 ± 15.4	33.9 ± 15.6	27.4 ± 13.9	−6.5 ± 16.9	26.5 ± 12.7	22.3 ± 11.7	−4.1 ± 13.8
Plant protein (g/day)	48.4 ± 17.36	43.7 ± 17.5	−4.6 ± 18.1	54.6 ± 18.0	48.7 ± 18.8	−5.9 ± 20.2	43.4 ± 15.0	39.7 ± 15.3	−3.6 ± 16.2
Total fat (g/day)	62.0 ± 22.6	56.2 ± 21.5	−5.76 ± 25.4	67.8 ± 23.2	60.8 ± 22.5	−7.0 ± 27.0	57.2 ± 20.8	52.5 ± 19.9	−4.7 ± 24.0
SFA (g/day)	22.6 ± 10.07	19.4 ± 9.2	−3.2 ± 10.9	24.7 ± 10.4	21.0 ± 9.6	−3.7 ± 11.5	21.0 ± 9.4	18.1 ± 8.6	−2.8 ± 10.4
PUFA (g/day)	11.1 ± 4.8	11.0 ± 5.4	−0.17 ± 5.8	12.0 ± 4.9	11.7 ± 5.6	−0.3 ± 6.1	10.4 ± 4.5	10.3 ± 5.1	−0.08 ± 5.6
MUFA (g/day)	17.6 ± 7.2	16.4 ± 7.1	−1.2 ± 8.65	19.2 ± 7.4	17.6 ± 7.4	−1.5 ± 9.1	16.4 ± 6.7	15.4 ± 6.7	−0.9 ± 8.1
Energy from carbohydrates (%)	64.9 ± 5.9	64.9 ± 6.1	−0.01 ± 7.1*	65.3 ± 5.7	65.3 ± 5.8	0.02 ± 6.88*	64.7 ± 6.0	64.6 ± 6.3	−0.05 ± 7.3*
Energy from protein (%)	13.2 ± 1.7	12.9 ± 1.8	−0.32 ± 2.1	13.3 ± 1.7	12.9 ± 1.8	−0.37 ± 2.1	13.1 ± 1.8	12.8 ± 1.8	−0.27 ± 2.1
Energy from fat (%)	23.5 ± 5.5	23.9 ± 5.8	0.4 ± 6.9	22.9 ± 5.2	23.3 ± 5.5	0.43 ± 6.5	24.0 ± 5.7	24.4 ± 6.0	0.37 ± 7.2

Abbreviations: MUFA, monounsaturated fatty acid; PUFA, polyunsaturated fatty acid; SFA, saturated fatty acid.

^a^ Pair's *t*‐test was used for changes in the two baseline and reassessment phases.

^b^ Unusual total energy intake (i.e., < 500 or > 3500 kcal/day for women and that is, < 800 or > 4200 kcal/day for men) was excluded from the analysis.

^c^ High‐quality carbohydrates and low‐quality carbohydrates.

* *p* > 0.05.

Regarding carbohydrate subtypes, the intake of low‐quality carbohydrates showed a greater absolute decline (22.3 ± 95.8 g/day) compared to high‐quality carbohydrates (14.1 ± 77.0 g/day). This reduction was more pronounced among men, with low‐quality carbohydrate intake decreasing by 28.5 ± 107.2 g/day, compared to 17.2 ± 85.1 g/day in women. However, the declines relative to baseline intake were similar for both low‐quality and high‐quality carbohydrates.

Among protein subtypes, animal protein intake demonstrated a larger absolute and relative reduction (5.1 ± 15.4 g/day and a 17% decline relative to baseline intake) than plant protein intake (4.6 ± 18.1 g/day and a 9.5% decline relative to baseline intake). In the fat subgroup analysis, saturated fatty acids (SFA) showed the greatest decrease (3.2 ± 10.9 g/day and a 14% decline relative to baseline intake). The relative reduction for monounsaturated fatty acids (MUFA) was 6.8%, while the corresponding value for polyunsaturated fatty acids (PUFA) was 1.5% (Table 2).

### 3.2 Carbohydrate

In the adjusted GEE model, each 1‐year increase in age was associated with a 1.19 g/day decrease in carbohydrate intake (*p* < 0.001). In addition, carbohydrate intake decreased by 39.91 g/day during the reassessment phase compared with baseline phase (*p* < 0.001). Women had a significantly lower carbohydrate intake compared with men, with a reduction of 82.91 g/day (*p* < 0.001). Individuals living in rural areas had 11.17 g/day higher carbohydrate intake than those in urban areas (*p* < 0.001). Regarding marital status, unmarried participants showed 17.60 g/day lower carbohydrate intake compared with married individuals (*p* < 0.001). Across socioeconomic status categories, all groups exhibited significantly lower carbohydrate intake compared with the lowest SES group (*p* < 0.01). Analysis of smoking status indicated that former smokers had 14.65 g/day higher carbohydrate intake compared with never smokers (*p* < 0.001). With respect to BMI, carbohydrate intake increased with higher body weight. Compared with normal‐weight individuals, underweight participants had 16.67 g/day lower carbohydrate intake (*p* < 0.001), whereas overweight and obese individuals showed 11.25 and 20.64 g/day higher carbohydrate intake, respectively (*p* < 0.001). Regarding morbidities, individuals with diabetes, hypertension, and cardiovascular disease had significantly 15.16, 5.16, and 8.49 g/day lower carbohydrate intake compared with their healthy counterparts, respectively (*p* < 0.001). In contrast, metabolic syndrome was associated with 4.57 g/day higher carbohydrate intake (*p* < 0.001) (Table 3).

**TABLE 3:** Multivariate GEE analysis of the association between independent variables and macronutrient and energy intake.

Variable^a^	Carbohydrate	*p*	Protein	*p*	Total fat	*p*	Total energy	*p*
	Coef (95% CI)^b^		Coef (95% CI)		Coef (95% CI)		Coef (95% CI)	
Age (year)	−1.19 (−1.34, −1.05)	**< 0.001**	−0.28 (−0.31, −0.25)	**< 0.001**	−0.23 (−0.26, −0.20)	**< 0.001**	−8.32 (−9.16, −7.49)	**< 0.001**
Time								
Baseline	Ref		Ref		Ref		Ref	
Reassessment	−39.91 (−41.36, −38.47)	**< 0.001**	−10.36 (−10.67, −10.06)	**< 0.001**	−5.74 (−6.04, −5.43)	**< 0.001**	−249.85 (−258.16, −241.53)	**< 0.001**
Gender								
Men	Ref		Ref		Ref		Ref	
Women	−82.91 (−85.41, −80.41)	**< 0.001**	−17.36 (−17.88, −16.85)	**< 0.001**	−9.15 (−9.62, −8.68)	**< 0.001**	−485.34 (−499.55, −471.12)	**< 0.001**
Residence type								
City	Ref		Ref		Ref		Ref	
Rural	11.17 (8.71, 13.64)	**< 0.001**	1.37 (0.87, 1.88)	**< 0.001**	1.57 (1.11, 2.04)	**< 0.001**	96.21 (72.47, 119.95)	**< 0.001**
Marital status								
Married	Ref		Ref		Ref		Ref	
Unmarried	−17.60 (−21.53, −13.68)	**< 0.001**	−3.18 (−3.98, −2.38)	**< 0.001**	−1.98 (−3.36, −0.61)	**< 0.001**	−98.83 (−121.20, −76.45)	**< 0.001**
Education								
Illiterate	Ref		Ref		Ref		Ref	
Up to Diploma	−2.29 (−4.72, 0.13)	0.065	−0.91 (−1.41, −0.42)	**< 0.001**	−1.20 (−1.71, −0.70)	0.946	−14.57 (−28.37, −0.76)	**0.039**
University education	−4.23 (−8.44, −0.02)	**0.049**	−0.82 (−1.70, 0.05)	0.065	−1.24 (−2.05, −0.43)	0.615	−52.11 (−91.61, −12.60)	**0.026**
Socioeconomic status								
The lowest	Ref		Ref		Ref		Ref	
Low	−4.72 (−7.48, −1.97)	**0.001**	0.87 (0.49, 1.24)	**< 0.001**	0.92 (0.42, 1.41)	**< 0.001**	−9.63 (−25.47934, 6.21)	0.234
Middle	−5.97 (−8.89, −3.06)	**< 0.001**	1.06 (0.66, 1.45)	**< 0.001**	1.85 (1.33, 2.37)	**< 0.001**	0.26 (−16.46, 17.00)	0.975
High	−4.98 (−8.09, −1.87)	**0.002**	1.59 (1.18, 2.00)	**< 0.001**	1.72 (1.18, 2.26)	**< 0.001**	9.55 (−8.31, 27.42)	0.295
The highest	−1.74 (−5.29, 1.80)	0.336	2.28 (1.80, 2.75)	**< 0.001**	3.39 (2.76, 4.02)	**< 0.001**	47.15 (26.78, 67.53)	**< 0.001**
Smoking status								
Never smoker	Ref		Ref		Ref		Ref	
Current smoker	5.53 (2.15, 8.91)	**< 0.001**	−0.42 (−0.91, 0.06)	0.092	−0.22 (−0.83, 0.38)	0.501	66.02 (33.16, 98.88)	**< 0.001**
Former smoker	14.65 (10.75, 18.55)	**< 0.001**	1.72 (0.89, 2.54)	**< 0.001**	1.94 (1.18, 2.70)	**< 0.001**	95.49 (67.03, 123.94)	**< 0.001**
Physical activity (MET)	−0.03 (−0.18, 0.10)	0.623	−0.01 (−0.02, 0.005)	0.191	−0.01 (0.06, 0.11)	**< 0.001**	0.70 (−0.10, 1.52)	0.090
Body mass index								
Normal	Ref		Ref		Ref		Ref	
Underweight	−16.67 (−24.02, −9.31)	**< 0.001**	−4.70 (−6.25, −3.28)	**< 0.001**	−3.14 (−4.52, −1.76)	**< 0.001**	−176.04 (−241.9, −110.12)	**< 0.001**
Overweight	11.25 (8.88, 13.62)	**< 0.001**	2.66 (2.17, 3.15)	**< 0.001**	1.72 (1.26, 2.18)	**< 0.001**	107.61 (86.11, 129.11)	**< 0.001**
Obesity	20.64 (17.90, 23.39)	**< 0.001**	4.82 (4.25, 5.39)	**< 0.001**	3.64 (3.10, 4.17)	**< 0.001**	195.62 (170.75, 220.49)	**< 0.001**
DMF	−0.12 (−0.24, −0.008)	**0.035**	−0.10 (−0.11, −0.08)	**< 0.001**	0.007 (−0.01, 0.02)	0.430	−0.77 (−1.43, −0.11)	**0.022**
Sleep duration (hour)	−0.54 (−0.79, −0.30)	**< 0.001**	−0.10 (−0.15, −0.05)	**< 0.001**	−0.15 (−0.27, −0.03)	0.139	−4.15 (−0.41, 8.72)	**0.002**
Hypertension								
No	Ref		Ref		Ref		Ref	
Yes	−5.16 (−8.17, −2.15)	**0.001**	−0.47 (−1.10, 0.14)	0.135	−1.06 (−1.62, −0.51)	**< 0.001**	−31.62 (−48.79, −14.44)	**< 0.001**
Diabetes								
No	Ref		Ref		Ref		Ref	
Yes	−15.16 (−18.10, −12.23)	**< 0.001**	−0.18 (−0.79, 0.42)	0.552	−0.71 (−1.25, −0.18)	**< 0.001**	−67.61 (−84.34, −50.88)	**< 0.001**
Cardiovascular disease								
No	Ref		Ref		Ref		Ref	
Yes	−8.49 (−11.41, −5.57)	**< 0.001**	−2.18 (−2.78, −1.58)	**< 0.001**	−2.11 (−2.64, −1.58)	**0.001**	−79.35 (−103.62, −55.09)	**< 0.001**
Metabolic syndrome								
No	Ref		Ref		Ref		Ref	
Yes	4.57 (2.05, 7.09)	**< 0.001**	0.50 (0.21, 0.80)	**0.001**	−1.14 (−1.53, −0.75)	**< 0.001**	17.12 (2.75, 31.49)	**0.020**

*Note:* Items with *p* < 0.05 highlighted in bold font; Items with *p* ≥ 0.05 are displayed in regular font.

^a^ Models were adjusted for age, time, gender, marital status, education, socioeconomic status (SES), body mass index (BMI), smoking, physical activity, DMF (decayed, missing, and filled) index, sleep duration, hypertension, diabetes, cardiovascular disease, metabolic syndrome, and interview center. Total energy intake was adjusted for macronutrients.

^b^ Coefficient (95% confidence interval).

### 3.3 Protein

In the adjusted GEE model, each 1‐year increase in age was associated with a 0.28 g/day decrease in protein intake (*p* < 0.001). In addition, protein intake decreased by 10.36 g/day during the reassessment phase compared with baseline phase (*p* < 0.001). Women had a significantly lower protein intake compared with men, with a reduction of 17.39 g/day (*p* < 0.001). Regarding marital status, unmarried participants had lower protein intake compared with married individuals, with a decrease of 3.18 g/day (*p* < 0.001). Individuals in higher SES groups consumed more protein compared with the lowest SES group. The increase in protein intake was 0.87 g/day in the low SES group, 1.06 g/day in the middle SES group, 1.59 g/day in the high SES group, and 2.28 g/day in the highest SES group (*p* < 0.001). Compared with normal‐weight individuals, protein intake was 4.70 g/day lower among underweight participants (*p* < 0.001), whereas it was higher among overweight and obese individuals, with increases of 2.66 and 4.82 g/day, respectively (*p* < 0.001). Independent of age and other variables, each additional unit of DMF was associated with a decrease of 0.10 g/day in protein intake (*p* < 0.001). Individuals with cardiovascular disease consumed less protein compared with those without the condition, with a decrease of 2.18 g/day (*p* < 0.001). In contrast, individuals with metabolic syndrome consumed more protein than those without, with an increase of 0.50 g/day (*p* = 0.001) (Table 3).

### 3.4 Total Fat

The results of the adjusted GEE model showed that each 1‐year increase in age was associated with a 0.23 g/day decrease in total fat intake (*p* < 0.001). In addition, total fat intake decreased by 5.74 g/day during the reassessment phase compared with baseline phase (*p* < 0.001). Women consumed an average of 9.15 g/day less fat compared with men during the reassessment phase (*p* < 0.001). Individuals living in rural areas consumed more fat compared with those living in urban areas, with an increase of 1.57 g/day (*p* < 0.001). Regarding marital status, unmarried participants had a lower fat intake than married individuals, with a decrease of 1.98 g/day (*p* < 0.001). Across SES categories, fat intake increased with higher SES. Compared with the lowest SES group, the increase in fat intake was 0.92 g/day in the low SES group, 1.85 g/day in the middle SES group, 1.72 g/day in the high SES group, and 3.39 g/day in the highest SES group (*p* < 0.001). Regarding BMI, compared with normal‐weight individuals, fat intake was lower in underweight individuals by 3.14 g/day, while it was higher in overweight and obese individuals by 1.72 and 3.64 g/day, respectively (*p* < 0.001). Participants with hypertension, diabetes, cardiovascular disease, and metabolic syndrome consumed less fat than healthy individuals, with decreases of 1.06, 0.71, 2.11, and 1.14 g/day, respectively (*p* < 0.001) (Table 3).

### 3.5 Total Energy

The results of the adjusted GEE model showed that each 1‐year increase in age was associated with an 8.32 kcal/day decrease in total energy intake (*p* < 0.001). In addition, total energy intake decreased by 249.85 kcal/day during the reassessment phase compared with the baseline phase (*p* < 0.001). Compared with men, women consumed 485.34 kcal/day less energy during the reassessment phase (*p* < 0.001). Participants living in rural areas had a higher total energy intake than those living in urban areas, with an increase of 96.21 kcal/day (*p* < 0.001). Unmarried individuals consumed 98.83 kcal/day less energy than married participants (*p* < 0.001). Compared with former smokers, current smokers consumed 95.49 kcal/day more energy (*p* < 0.001). In addition, compared with individuals with no formal education, those with university education consumed 52.11 kcal/day less energy (*p* = 0.026). Regarding BMI, overweight and obese participants consumed 107.61 and 195.62 kcal/day more energy than normal‐weight individuals, respectively (*p* < 0.001). Individuals with hypertension, diabetes, and cardiovascular disease had significantly lower total energy intake compared with healthy participants, with decreases of 31.62, 67.61, and 79.35 kcal/day, respectively (*p* < 0.001). In contrast, participants with metabolic syndrome had a significantly higher total energy intake by 17.12 kcal/day (*p* = 0.020) (Table 3).

Considering the multicenter nature of the PERSIAN study, the results regarding the effects of different centers on macronutrients and total energy are presented in Table S2.

### 3.6 Changes in Macronutrients in Different Centers

Compared to the residents of Ravansar in the western part of Iran, Hovizeh in Khuzestan province experienced the highest increase in carbohydrate intake, while Urmia had the lowest increase over the 6‐year period. Regarding protein intake, Zahedan showed the highest increase, whereas the Azar cohort in the northwest of Iran experienced the greatest decrease (Table S2).

In terms of total fat intake, Hovizeh had the most significant decrease. While all other centers reported a decline in fat consumption, the people in the Azar cohort did not show significant changes compared to Ravansar. For total energy intake, Guilan experienced the largest decrease, while Fasa had the largest increase (Table S2).

### 3.7 Assessment of Interaction Effects and Gender‐Stratified Analyses

In the fully adjusted GEE models, the interaction term between time (reassessment vs. baseline) and socioeconomic status was not statistically significant for total energy or any of the macronutrients (carbohydrate, protein, and total fat) (*p* > 0.05). In contrast, the time × gender interaction was statistically significant for all outcomes (*p* < 0.05), indicating that the magnitude of change in dietary intake over the 6‐year period differed between men and women (men had a larger decrease over time). Although the results of interaction terms have not been presented in Table 3 (because of no changes in other coefficients presented in Table 3), Table 2 clearly show such differences in declines between men and women.

## 4 Discussion

This study examines changes in macronutrient intake between the two phases of the PERSIAN cohort study (baseline and reassessment phase) over a 6‐year period (2015–2021). The findings of this study indicate that the daily intake of macronutrients in the studied population has decreased during the reassessment phase. This reduction is observed in total energy, carbohydrates, protein, and total fat. Notably, there has been a decrease of 250.1 kcal in daily energy intake, along with a significant reduction in carbohydrate and protein consumption, raising concerns about the dietary quality of the population. Additionally, the percentage of energy derived from carbohydrates and protein has also declined. It is noteworthy that the population has aged over this period, with the average age increasing from 47.5 to 54.5 years. This demographic change has partly led to alterations in dietary habits and health‐related outcomes as well as other related factors.

### 4.1 Nutritional Changes Review

The present results differ from the findings of the Aghayan et al. (2020), who conducted a comprehensive analysis of dietary patterns among Iranian adults within the framework of the Tehran Lipid and Glucose Study (TLGS) from 2006 to 2017. They found that during this study period, there was an increase in carbohydrate and protein intake, which is inconsistent with the results of our study (Aghayan et al. 2020). In a study by Heidari et al. (2019) involving individuals over 40 years old in Iran, the results indicated that the energy contribution from carbohydrate, protein and fat were 59.9%, 17.4%, and 25.7%. These findings slightly differ from the results of the present study (Heidari et al. 2019). Moreover, in the study by Noormohammadi et al. (2022), conducted on 723 individuals aged 20–64 years in the city of Urmia, Iran, the mean total energy intake was reported as 2920 kcal. The mean intake of protein, carbohydrates, and total fat in this population was 111.3, 426.1, and 93.1 g, respectively. These values were higher than those observed in the present study and did not correspond with our findings (Noormohammadi et al. 2022). The findings of the study by Al Hourani et al. (2023) conducted in Jordan between October 2021 and March 2022 in a population aged 8 years and older, indicated that adult macronutrient intake in Jordan, except for carbohydrate, was substantially higher than that observed in the present study. They reported mean total energy, carbohydrate, protein, and total fat intakes of 2559 kcal, 301, 98.5, and 110 g, respectively, whereas in the present study, the corresponding values were 2128 kcal, 346, 69, and 56 g. This is not surprising as the dietary habits in Iran is different from other countries and they included wider age groups than our study (Al Hourani et al. 2023). In a study conducted by Ahmed et al. (2022) on the population over 18 years old in Bangladesh, it was observed that between 2011 and 2018, there were reductions in energy intake (3%–8%), protein (3%–9%), and carbohydrates (9%–16%) across all age groups, which is consistent with our observed trends in our study (Ahmed et al. 2022). Additionally, in a systematic review conducted by Doggui et al. (2021) examining macronutrient intake in the Eastern Mediterranean Region, the included studies spanned the period from 2012 to 2020. The review reported that the average energy intake of Iranians was 2166.3 kcal, almost the same as our findings, which is lower than that of Jordan, Lebanon, Libya, Palestine, Tunisia, and the UAE. The average protein intake of Iranians in our study was 68.7 g/day, which is lower than the overall average intake in the region of around 80 g/day (Doggui et al. 2021). In another study examining changes in food consumption and nutritional intake in Kazakhstan from 2001 to 2018, the results showed that per capita energy consumption increased significantly to 3716 kcal/day, representing a 31.5% growth, over the period of study. Additionally, protein intake experienced a surge of 103.87%, while fat consumption increased by 78.37%. These findings indicate fundamental changes in the dietary patterns and lifestyle of Kazakhstan's population during this period (Jia et al. 2022). Interestingly, Kazakhstan's per capita energy intake has even exceeded the recommendation of the EAT‐Lancet Commission (EAT), which suggests 2500 kcal/day (Willett et al. 2019).

The study by Saito et al. (2018) revealed notable nutritional trends in the Japanese population aged 20–79 years between 1995 and 2016. They found a decrease in total energy intake with an annual percentage change of 0.5% and a reduction in energy proportion from protein by 3% (Saito et al. 2018). As a result, Japan, by balancing daily energy intake from food sources, is recognized as one of the countries with the lowest prevalence of overweight or obesity (Collaboration NRF 2016). However, the trend of total energy intake and macronutrients over time in other developed countries, such as the United States (Yancy et al. 2014), Germany (Gose et al. 2016), France (Dubuisson et al. 2010), the United Kingdom (Whitton et al. 2011), Australia (Bel et al. 2019), China (Huang et al. 2023), Belgium (Bel et al. 2019), and South Korea (Yun et al. 2017), has either remained stable or increased, which is inconsistent with the results of this study.

In addition to the overall decline in total macronutrient intake, reductions were observed across all examined nutrient subtypes, including high‐ and low‐quality carbohydrates, animal and plant protein, and different types of dietary fats. The greater reduction in low‐quality carbohydrates (in absolute terms) and animal protein (in both absolute and relative terms) may reflect changes in food affordability and dietary choices during periods of economic instability. Among fat subtypes, saturated fatty acids (SFA) showed the largest decline over time. From a nutritional perspective, this finding may be considered potentially beneficial, as higher SFA intake has been associated with an increased risk of cardiovascular disease and metabolic disorders (Aramburu et al. 2024). Nevertheless, the simultaneous reduction in MUFA and PUFA intake (although they show lower declines in both absolute and relative terms) suggests that these changes were not necessarily due to improved dietary quality or healthier food choices. Rather, the observed pattern likely reflects a generalized reduction in overall food intake and dietary diversity, possibly driven by economic constraints, rising food prices, and reduced purchasing power during the study period. These socioeconomic and structural factors influencing dietary changes are discussed in greater detail in Section 4.3.

### 4.2 Factors Influencing Nutritional Changes

As individuals age, their nutritional habits are shaped by various factors, which can lead to positive or negative changes in health status (Tabiee et al. 2024). Most studies indicate that in the relationship between food intake and gender, women across all age groups (except during pregnancy and breastfeeding) have a higher prevalence of inadequate nutrient intake compared to men for almost all nutrients (Ratsavong et al. 2020; Park et al. 2018; Bertrand et al. 2021). Due to their roles as caregivers, women often prioritize feeding their families over themselves (Bouapao et al. 2016). Moreover, in many cultures, men typically eat first, and women may possess less knowledge about nutritional requirements (Johnson et al. 2018). A few decades ago, it was recognized that dietary habits, including the food choices of populations, are significantly influenced by geographic regions and residential locations (Grande Covián et al. 2014; Culebras 2014). In the study by Samaniego‐Vaesken et al. (2018) in Spain, which analyzed the differences in the behavior of rural and urban consumers, they found that different price levels in each type of locality are the main factor for notable differences in food purchasing patterns. They also emphasized that income level is only considered a determining factor in food choices when comparing rural and urban settings (Samaniego‐Vaesken et al. 2018). Numerous other studies have emphasized the critical role of SES in shaping food choices (Ahmed et al. 2022; Abdollahi et al. 2016; Spronk et al. 2014; Sobhani et al. 2022; Guiné et al. 2020). The present study demonstrates that energy, protein, and fat intake are significantly higher in higher socioeconomic classes compared to those in lower classes. Conversely, carbohydrate consumption is higher in lower socioeconomic classes. These findings are consistent with other studies (Wimmer et al. 2023; Sakurai et al. 2018). These patterns may reflect differential access to diverse and nutrient‐dense foods, as higher socioeconomic groups are more likely to afford protein‐rich and high‐quality fat sources such as meat, dairy, and nuts. In contrast, lower socioeconomic groups often rely on more affordable, carbohydrate‐dense staple foods, which may contribute to an imbalanced dietary profile. Such disparities highlight the importance of targeted nutritional policies and interventions aimed at improving diet quality among lower socioeconomic groups (Carrasco‐Marín et al. 2024). This pattern has also been reported in recent studies showing that lower socioeconomic status is associated with poorer diet quality and lower adherence to healthy dietary recommendations (Wang et al. 2024; McCullough et al. 2022).

Given the significant impact of obesity on public health, it is essential to examine the changes in intake of macronutrients among such group. In fact, a thorough analysis of macronutrient intake among obese and overweight individuals in the Iranian population is of particular importance (Rashidi et al. 2018). In a study conducted by Rashidi et al. (2018) in Iran, it was shown that overweight and obesity do not have a significant relationship with carbohydrates and protein intake. The results of this study were not consistent with the findings of the present study and other reports (Rashidi et al. 2018; Salehi et al. 2024; Zhao et al. 2020). One possible explanation for this discrepancy may be the longitudinal nature of the current study, in which the mean age of participants increased from 47.5 to 54.5 years during follow‐up. Aging is associated with alterations in metabolism, body composition, appetite regulation, and dietary behaviors, which may influence macronutrient and total energy intake (Kassis et al. 2023; Ou et al. 2022). Furthermore, overweight and obese participants in the present study had significantly higher intakes of carbohydrates, protein, fat, and total energy, supporting evidence that excessive energy intake and consumption of energy‐dense foods contribute to obesity development (Harb et al. 2023).

In the study by Motamed et al. (2019), conducted on a population aged 35–68 years in Mashhad, Iran, men with metabolic syndrome consumed more protein compared to men without the condition, averaging 71.4 g/day versus 65.9 g/day, which is higher than that observed in the present study. However, the difference in protein intake among women was not statistically significant, and no other macronutrients showed significant differences (Motamed et al. 2019). Similarly, in Iranian cohorts such as the Tehran Lipid and Glucose Study and the Shahedieh cohort, macronutrient quality has generally been associated with a lower risk of metabolic syndrome (Farhadnejad et al. 2024; Sadeghi et al. 2021). However, among affected individuals, higher protein intake patterns—particularly in those with diabetes and hypertension—have been observed, which may reflect dietary adaptations following diagnosis (Sadeghi et al. 2021).

The relationship between macronutrient intake and chronic diseases (type 2 diabetes, cardiovascular diseases, and high blood pressure) was also examined in this study. In the study by Oosterwijk et al. (2022), conducted on patients with type 2 diabetes in the Netherlands, it was found that 83% of participants consumed protein at or below the recommended intake (0.8 g/kg/day) (Oosterwijk et al. 2022). However, the results of the present study showed that there was no statistically significant association between diabetes and daily protein intake. Similarly, cardiovascular disease has also been investigated in relation to macronutrient intake. In the study by Beheshti et al. (2024), data were extracted from the Prospective Epidemiologic Research Studies in Iran (PERSIAN) cohort in Sabzevar, Iran, including 1535 patients with cardiovascular disease and 2706 individuals without cardiovascular disease. The results showed that cardiovascular patients consumed an average of 62 g/day of protein, compared with 63 g/day in healthy individuals. Daily energy intake was 2392 kcal in cardiovascular patients versus 2541 kcal in healthy controls. Despite lower protein and total energy intake, cardiovascular patients consumed slightly more carbohydrates per day than healthy individuals (331 g vs. 328 g). These findings were not consistent with the results observed in the present study (Beheshti et al. 2024). In the study by Alissa and Alama (2015), conducted in Saudi Arabia as a case–control study, it was found that patients with cardiovascular disease had lower total energy intake (1533 kcal vs. 1864 kcal) and lower macronutrient intake compared to the control group (carbohydrates: 191–200 g/day vs. 212 g/day, protein: 57–63 g/day vs. 66 g/day, and total fat: 63–69 g/day vs. 82 g/day). The authors attributed these differences to cardiac patients' awareness of the impact of dietary modifications on their disease (Alissa and Alama 2015). These findings were consistent with the results of the present study.

This study analyzed the consumption of macronutrients and total energy at various centers of the PERSIAN cohort. The results indicate that over 6 years, the consumption of macronutrients and total energy has decreased. Regarding carbohydrates, most centers showed an increase in consumption compared to the reference center, while a decrease in consumption was observed for protein, fat, and total energy. Ethnic diversity (Arab, Lor, Kurd, Turk, etc.) and environmental and socioeconomic differences at each center can influence their consumption patterns (Poustchi et al. 2018). Therefore, these factors, along with other reasons that will be discussed later, have contributed to the decrease in these intakes.

### 4.3 Economic and Structural Explanations for Macronutrient Decline

To understand the reasons behind the results observed in the studied population, we must point to the other national factors that influence changes in nutrient consumption, those that we did not measure directly. The economic status and individual income, the rising prices of various food items, personal preferences and beliefs, cultural traditions, as well as geographical, environmental, and social factors all play significant roles in food choices. The increase in food prices, coupled with the disparity between the inflation rate and the growth of household income, significantly impacts dietary consumption and food security (Meade and Thome 2017; Mollarasouli et al. 2024). From 2015 to 2021, the inflation rate in several provinces involved in the PERSIAN cohort, including Kerman, Yazd, Ilam, and Shahrekord, exceeded 50%. Additionally, both the unemployment rate and the Gini coefficient rose substantially during this period (Hejazi and Emamgholipour 2020; Iran SCo 2023). In January 2023, the Consumer Price Index for households in the country (with a base year of 2016) reached 608.0, indicating an average price increase of 608% compared to 2016 (Hejazi and Emamgholipour 2020; Iran SCo 2023). During this period, Iran's gross domestic product (GDP) significantly decreased by more than 20% (Gharehgozli 2017). The Gini coefficient has shown a notable increase since 2012, reaching over 0.40 (Noferesti and Varahrami 2023). In addition, fluctuations in the US dollar exchange rate have significantly increased in recent years, serving as one of the key factors in determining the prices of goods and services. This increase in the US dollar exchange rate has negatively impacted the purchasing power of households, consequently affecting their food consumption patterns. Therefore, the government has implemented cash subsidy programs to support vulnerable segments of society. These subsidies are designed to alleviate financial pressures on low‐income households, helping them cope with rising living costs and ensuring access to essential goods and services. In conclusion, dietary changes driven by economic and social factors highlight the serious challenges faced by households. These conditions not only affect individuals' health and nutrition but may also exacerbate social and economic inequalities.

## 5 Strengths and Limitations

The PERSIAN study is a longitudinal population‐based study and is the first research to examine the nutritional status in the PERSIAN cohort. This study has a sufficient sample size of 32,220 individuals. Given the ethnic diversity in Iran, which includes Persians, Turks/Azeris, Kurds, Lurs, Baluchis, Arabs, and others, samples were selected from 18 distinct regions across Iranian provinces. This selection aims to depict a wide range of environmental diversity, lifestyle variations, and socioeconomic differences, as well as various exposures that influence outcome patterns. The data for this study were collected in two time periods, 6 years apart. Although the number of nutritional measurements is limited to two times, the study period coincided with several economic crises in Iran, leading to a worsening of the SES of the Iranian population. Using the same FFQ questionnaire that has been previously validated strengthens our ability to make robust comparisons (Najafi et al. 2022). The assessment of changes in food consumption was conducted at the level of macronutrients and total energy, which can provide a more comprehensive picture of the changes that have occurred. However, in assessing dietary intake using FFQ, like other methods, there are biases such as measurement error, including underreporting or over reporting of some food items or the total intake due to recall bias. Nutritional status varies across different provinces of Iran due to socioeconomic influences and cultural habits. These differences can affect access to food and dietary choices. However, the sample examined in this study may not be a complete representative of the nutritional status of the entire population of Iran and may not accurately reflect the dietary habits of the whole country. The observational nature of the study inherently limits causal inferences; observed changes in macronutrient intake cannot be definitively attributed to economic factors, disease status, or other exposures, as residual confounding, reverse causation (e.g., individuals with chronic conditions altering diet post‐diagnosis), or unmeasured variables may explain findings.

## 6 Conclusion

This study reveals significant reductions in macronutrient and overall energy consumption among Iranian adults during the periods of 2015–2017 and 2021–2023, reflecting a reduction of 250 kcal/day in energy intake, accompanied by reductions in carbohydrates (40.3 g/day), proteins (10.3 g/day), and fats (5.7 g/day). These alterations, driven primarily by economic pressures such as elevated inflation rates, diminished purchasing capacity, and food scarcity resulting from different reasons, were influenced by variables including gender (with females exhibiting more pronounced declines in protein intake), geographic location (rural vs. urban areas), SES, BMI, and the prevalence of noncommunicable diseases. These trends highlight a phenomenon termed economically imposed caloric restriction wherein numerical limitations are imposed without accompanying improvements in quality, thereby potentially intensifying the risks of malnutrition amidst a continued dependency on refined carbohydrates.

To effectively respond to these shifts in dietary patterns and to enhance overall nutritional status, the following strategic interventions may be beneficial: (1) Protein vouchers or subsidized nutritional supplements tailored specifically for at‐risk people, including women, the elderly, and households with low socioeconomic status; (2) Initiate comprehensive nationwide public health initiatives advocating for affordable, nutrient‐dense food alternatives within the financial means of families; and (3) Initiating policies that can ultimately improve the overall economic situation will lead to better socioeconomic conditions in the country, which in turn will enhance the nutritional status of the Iranian population. The effectiveness of these interventions, assessed through periodic surveys and other longitudinal studies, has the potential to reduce the risks associated with diet‐related noncommunicable diseases and to promote equitable food security throughout Iran.

## Author Contributions

**Farid Najafi:** data curation, writing – review and editing, writing – original draft, funding acquisition, investigation, conceptualization, methodology, validation, visualization, software, formal analysis, project administration, supervision, resources. **Neda Izadi:** conceptualization, methodology, formal analysis, investigation, writing – review and editing. **Shahab Rezaeian:** conceptualization, investigation, methodology, formal analysis, writing – review and editing, data curation. **Yahya Pasdar:** conceptualization, investigation, methodology, formal analysis, writing – review and editing, data curation. **Fatemeh Khosravi Shadmani:** conceptualization, investigation, methodology, writing – review and editing, formal analysis, data curation. **Amir Bagheri:** conceptualization, investigation, methodology, writing – review and editing, formal analysis. **Hossein Poustchi:** data curation, investigation. **Sareh Eghtesad:** conceptualization, formal analysis, writing – review and editing, data curation. **Farhad Pourfarzi:** writing – review and editing, data curation. **Azim Nejatizadeh:** data curation, writing – review and editing. **Mojtaba Farjam:** data curation, writing – review and editing. **Farahnaz Joukar:** data curation, writing – review and editing. **Amir Hoshang Bavarsad:** data curation, writing – review and editing. **Mohammad Reza Fattahi:** data curation, writing – review and editing. **Motahareh Kheradmand:** data curation, writing – review and editing. **Ali Ahmadi:** data curation, writing – review and editing. **Mohammad Hossein Somi:** data curation, writing – review and editing. **Iraj Mohebbi:** data curation, writing – review and editing. **Sayed Bahman Panahande:** data curation, writing – review and editing. **Hassan Mozaffari‐Khosravi:** data curation, writing – review and editing. **Alireza Ansari‐Moghadam:** data curation, writing – review and editing. **Masoumeh Ghoddusi Johari:** data curation, writing – review and editing. **Habib Allah Shahryari:** conceptualization, investigation, writing – original draft, methodology, validation, visualization, writing – review and editing, formal analysis, software, data curation, resources.

## Funding

This research was supported by Kermanshah University of Medical Sciences (Grant No. 92472), and the National Institute for Medical Research Development (NIMAD) (Grant No. 4020979). The Iranian Ministry of Health and Medical Education (MOHME), Iran also contributed to the funding of the PERSIAN Cohort study (Grant No. 700/534).

## Ethics Statement

While each cohort center received the ethical approval from local universities, for the purpose of this study and pooling all PERSIAN data, the ethics committee of Kermanshah University of Medical Sciences approved the study (IR.KUMS.REC.1403.095).

## Conflicts of Interest

The authors declare no conflicts of interest.

## Supporting information


**Table S1:** Univariate GEE analysis of the association between independent variables and macronutrient and energy intake.
**Table S2:** Multivariate GEE analysis of the associations between Interview center variables with macronutrient intake and total energy intake.

## Data Availability

The data analyzed in the study are available from the corresponding author upon reasonable request.
