# Supplementary material for: Nutritional Changes and Contributing Factors in Iran: A Comprehensive Study From PERSIAN Cohort Study (2015–2017 and 2021–2023)
Source: Food Sci Nutr. 2026 Jun 25;14(6):e72060. doi: 10.1002/fsn3.72060 (PMC13305144; doi:10.1002/fsn3.72060)
Supplement: Supplementary file 1 — Table S1: Univariate GEE analysis of the association between independent variables and macronutrient and energy intake. Table S2: Multivariate GEE analysis of the associations between Interview center variables with macronutrient intake and total energy intake. [file FSN3-14-e72060-s001.docx]

**Supplemental Page**

**Table 1: Univariate GEE analysis of the association between independent variables and macronutrient and energy intake**

| **Variable** | | **Carbohydrate**  **Coef (95%CI)** ^†^ | **P value** | **Protein**  **Coef (95%CI)** ^†^ | **P value** | **Total fat**  **Coef (95%CI)** ^†^ | **P value** | **Total Energy**  **Coef (95%CI)** ^†^ | **P value** |
| --- | --- | --- | --- | --- | --- | --- | --- | --- | --- |
| **Age (year)** | | -0.70 (-0.84, -0.55) | <0.001 | -0.23 (-0.26, -0.20) | <0.001 | -0.29(-0.32, -0.26) | <0.001 | -6.52(-7.38, -5.67) | <0.001 |
| **Gender** | |  |  |  |  |  |  |  |  |
|  | Men | Ref |  | Ref |  | Ref |  | Ref |  |
|  | Women | -89.58 (-91.98, -87.17) | <0.001 | -18.7(-19.25, -18.23) | <0.001 | -10.56(-11.04, -10.07) | <0.001 | -527.73(-541.52, -513.95) | <0.001 |
| **Time** |  |  |  |  |  |  |  |  |  |
|  | Baseline | Ref |  | Ref |  | Ref |  | Ref |  |
|  | Reassessment | -40.34(-41.67, -39.01) | <0.001 | -10.03(-10.31, -9.75) | <0.001 | -5.76(-6.04, -5.48) | <0.001 | -250.15(-257.80, -242.50) | <0.001 |
| **Residence Type** | |  |  |  |  |  |  |  |  |
|  | City | Ref |  | Ref |  | Ref |  | Ref |  |
|  | Rural | 11.72 (9.32, 14.12) | <0.001 | -0.02(-0.52, 0.47) | 0.931 | -0.11(-0.55, 0.31) | 0.113 | 52.81(38.97, 66.65) | <0.001 |
| **Marital Status** | |  |  |  |  |  |  |  |  |
|  | Married | Ref |  | Ref |  | Ref |  | Ref |  |
|  | Unmarried | -53.67 (-57.66, -49.68) | <0.001 | -11.21(-12.05,-10.38) | <0.001 | -7.62 (-8.37, -6.86) | <0.001 | -323.23 (-346.32, -300.15) | <0.001 |
| **Education** | |  |  |  |  |  |  |  |  |
|  | Illiterate | Ref |  | Ref |  | Ref |  | Ref |  |
|  | Up to Diploma | 30.34(28.14, 32.55) | <0.001 | 7.71(7.24, 8.17) | <0.001 | 5.98(5.56, 6.40) | <0.001 | 200.53(187.79, 213.26) | <0.001 |
|  | University Education | 39(35.13, 42.94) | <0.001 | 11.31(10.49,12.12) | <0.001 | 8.35(7.63, 9.06) | <0.001 | 263.67(241.20, 286.14) | <0.001 |
| **Socioeconomic Status** | |  |  |  |  |  |  |  |  |
|  | The poorest | Ref |  | Ref |  | Ref |  | Ref |  |
|  | Poor | 2.82 (.02, 5.63) | 0.048 | 2.54 (1.95, 3.14) | <0.001 | 2.05(1.51, 2.59) | <0.001 | 35.2(19.00, 51.44) | <0.001 |
|  | Middle | 5.85 (2.96, 8.74) | <0.001 | 4.15 (3.54, 4.76) | <0.001 | 4.42(3.87, 4.97) | <0.001 | 71.35(54.66, 88.04) | <0.001 |
|  | Rich | 9.08 (6.14, 12.01) | <0.001 | 5.37 (4.76, 5.99) | <0.001 | 5.29(4.74, 5.85) | <0.001 | 93.83(76.89, 110.78) | <0.001 |
|  | The Richest | 12.93 (9.78, 16.07) | <0.001 | 7.28 (6.63, 7.95) | <0.001 | 7.78(7.19, 8.37) | <0.001 | 132.5(114.37, 150.69) | <0.001 |
| **Smoking Status** | |  |  |  |  |  |  |  |  |
|  | Never smoker | Ref |  | Ref |  | Ref |  | Ref |  |
|  | Current smoker | 41.18(38.22, 44.15) | <0.001 | 6.79(6.16, 7.42) | <0.001 | 4.39(3.81, 4.96) | <0.001 | 231(213.87, 248.16) | <0.001 |
|  | Former smoker | 75.10(71.72, 78.48) | <0.001 | 14.64(13.93,15.36) | <0.001 | 9.20(8.55, 9.86) | <0.001 | 441.7(422.19, 461.31) | <0.001 |
| **Physical Activity (MET)** | | 1.56 (1.39, 1.74) | <0.001 | 0.29(0.25, 0.33) | <0.001 | 0.28(0.25, 0.32) | <0.001 | 10(9.01, 10.99) | <0.001 |
| **Body Mass Index** | |  |  |  |  |  |  |  |  |
|  | Normal | Ref |  | Ref |  | Ref |  | Ref |  |
|  | Underweight | -2.99 (-10.73, 4.74) | 0.449 | -3.50 (-4.67, -1.42) | <0.001 | -1.64 (-3.10, -.18) | 0.027 | -35.16(-79.90, 9.58) | 0.123 |
|  | Overweight | -1.56 (-3.98, 0.86) | 0.208 | 0.61 (-0.04, 1.27) | 0.021 | 0.24 (-0.21, 0.70) | 0.295 | -4.23(-18.27, 9.80) | 0.554 |
|  | Obesity | -10.27 (-12.98, -7.57) | <0.001 | -0.90 (-1.46, -0.33) | 0.002 | -0.37 (-0.87, 0.12) | 0.144 | -51.56(-67.17, -35.96) | <0.001 |
| **DMF (Number of teeth)** | | -0.08 (-0.21, 0.05) | 0.239 | -0.15 (-0.17, -0.12) | <0.001 | -0.05 (-0.08, -0.02) | <0.001 | -1.38(-2.15, -0.60) | <0.001 |
| **Sleep Duration (Hour)** | | -1.96 (-2.28, -1.70) | <0.001 | -0.45(-0.51, -0.39) | <0.001 | -0.25 (-0.31, -0.20) | <0.001 | -11.31(-12.96, -9.65) | <0.001 |
| **Hypertension** | |  |  |  |  |  |  |  |  |
|  | No | Ref |  | Ref |  | Ref |  | Ref |  |
|  | Yes | -18.55(-21.47, -15.63) | <0.001 | -3.51 (-4.13, -2.89) | <0.001 | -5.36(-5.92, -4.80) | <0.001 | -137.6(-154.39, -120.85) | <0.001 |
| **Diabetes** | |  |  |  |  |  |  |  |  |
|  | No | Ref |  | Ref |  | Ref |  | Ref |  |
|  | Yes | -25.66 (-29.10, -22.23) | <0.001 | -2.30 (-3.03, -1.57) | <0.001 | -4.12 ( -4.78, -3.46) | <0.001 | -149.72(-169.53, -129.92) | <0.001 |
| **Cardiovascular Disease** | |  |  |  |  |  |  |  |  |
|  | No | Ref |  | Ref |  | Ref |  | Ref |  |
|  | Yes | -28.67(-31.57, -25.77) | <0.001 | -5.80(-6.42, -5.18) | <0.001 | -6.66(-7.22, -6.11) | <0.001 | -199.6(-216.33, -182.99) | <0.001 |
| **Metabolic Syndrome** | |  |  |  |  |  |  |  |  |
|  | No | Ref |  | Ref |  | Ref |  | Ref |  |
|  | Yes | -10.27 (-12.93, -7.61) | <0.001 | -1.01 (-1.58, -0.45) | <0.001 | -3.66 (-4.17, -3.15) | <0.001 | -79.8(-95.17, -64.59) | <0.001 |
| **Interview Center** | |  |  |  |  |  |  |  |  |
|  | Ravansar | Ref |  | Ref |  | Ref |  | Ref |  |
|  | Guilan | -81.52(-88.94, -74.10) | <0.001 | -26.57(-28.13, -25.0) | <0.001 | -31.07(-32.43, -29.71) | <0.001 | -710.63(-754.15, -667.11) | <0.001 |
|  | Fasa | 77.20(69.76, 84.64) | <0.001 | 1.89(.32, 3.46) | 0.018 | -11.87(-13.24, -10.50) | <0.001 | 201.94(158.28, 245.59) | <0.001 |
|  | Azar | 22.98(13.22, 32.74) | <0.001 | -5.23(-7.29, -3.17) | <0.001 | 2.10(.31, 3.89) | 0.021 | 52.53(-4.73, 109.80) | 0.072 |
|  | Kharameh | -14.37(-21.86, -6.89) | <0.001 | -15.71(-17.29, -14.1) | <0.001 | -19.14(-20.51, -17.77) | <0.001 | -295.44(-339.36, -251.51) | <0.001 |
|  | Mazandaran | -59.18(-68.27, -50.09) | <0.001 | -22.02(-23.94, -20.1) | <0.001 | -23.02(-24.69, -21.35) | <0.001 | -560.29(-613.60, -506.97) | <0.001 |
|  | Zahedan | 14.17(6.17, 22.16) | <0.001 | 4.63(2.94, 6.32) | <0.001 | -11.12(-12.59, -9.65) | <0.001 | -28.78(-75.71, 18.13) | 0.229 |
|  | Yazd | -40.83(-52.05, -29.60) | <0.001 | -20.53(-22.90, -18.1) | <0.001 | -26.61(-28.67, -24.55) | <0.001 | -493.57(-559.41, -427.73) | <0.001 |
|  | Rafsanjan | -92.67(-101.31, -84.03) | <0.001 | -23.42(-25.24, -21.6) | <0.001 | -24.52(-26.11, -22.93) | <0.001 | -682.83(-733.52, -632.14) | <0.001 |
|  | Hoveizeh | 102.49(95.02, 109.97) | <0.001 | 4.17(2.59, 5.75) | <0.001 | -19.11(-20.48, -17.73) | <0.001 | 251.61(207.76, 295.46) | <0.001 |
|  | Shahrekord | -1.61(-9.93, 6.70) | 0.704 | -1.41(-3.17, .33) | 0.114 | -8.80(-10.33, -7.27) | <0.001 | -94.47(-143.27, -45.66) | <0.001 |
|  | Bandare Kong | -35.94(-44.32, -27.57) | <0.001 | -7.14(-8.91, -5.37) | <0.001 | -9.45(-10.99, -7.91) | <0.001 | -277.74(-326.87, -228.61) | <0.001 |
|  | Urmia Lake | -62.73(-71.06, -54.39) | <0.001 | -15.78(-17.54, -14.0) | <0.001 | -14.68(-16.21, -13.14) | <0.001 | -448.79(-497.72, -399.87) | <0.001 |
|  | Ardabil | 6.65(-.91, 14.22) | 0.085 | -2.46(-4.05, -.86) | <0.001 | -9.15(-10.54, -7.76) | <0.001 | -74.46(-118.86, -30.05) | <0.001 |
|  | Sabzevar | -23.60(-33.13, -14.07) | <0.001 | -17.11(-19.13, -15.1) | <0.001 | -18.22(-19.97, -16.47) | <0.001 | -337.84(-393.75, -281.93) | <0.001 |
|  | Dena | 5.70(-3.53, 14.94)  -66.56(-74.93, -58.19) | 0.226  <0.001 | -9.11(-11.06, -7.16)  -21.66(-23.42, -19.8) | <0.001 | -9.19(-10.88, -7.49)  -26.35(-27.88, -24.81) | <0.001 | -115.66(-169.85, -61.46)  -597.14(-646.26, -548.03) | <0.001 |
|  | Kavar |  |  |  | <0.001 |  | <0.001 |  | <0.001 |

^†^_Coefficient (95% Confidence Interval)_

**Table 2:** Multivariate GEE analysis of the associations between Interview center variables with macronutrient intake and total energy intake

| **Variable**^†^ | | | **Carbohydrate**  **Coef (95%CI)** ^††^ | **P value** | | | **Protein**  **Coef (95%CI)** | **P value** | | | **Total fat**  **Coef (95%CI)** | **P value** | | **Total Energy**  **Coef (95%CI)** | **P value** | |
| --- | --- | --- | --- | --- | --- | --- | --- | --- | --- | --- | --- | --- | --- | --- | --- | --- |
| **Interview Center** | |  | | |  |  | | |  |  | | |  |  | |  |
|  | Ravansar | Ref | | |  | Ref | | |  | Ref | | |  | Ref | |  |
|  | Guilan | 33.65 (31.63, 35.681) | | | <0.001 | -3.42 (-4.04, -2.80) | | | <0.001 | -12.32 (-13.16, -11.47) | | | <0.001 | -641.38 (-682.17, -600.5) | | <0.001 |
|  | Fasa | 41.34 (39.23, 43.45) | | | <0.001 | -2.88 (-3.53, -2.24) | | | <0.001 | -16.06 (-16.94, -15.17) | | | <0.001 | 253.4 (210.47, 296.42) | | <0.001 |
|  | Azar | 13.08 (10.46, 15.71) | | | <0.001 | -6.75(-7.55, -5.94) | | | <0.001 | 0.53(-.56, 1.62) | | | 0.343 | 100.13 (46.56, 153.70) | | <0.001 |
|  | Kharameh | 31.77 (29.72, 33.82) | | | <0.001 | -4.91 (-5.53, -4.28) | | | <0.001 | -10.87 (-11.73, -10.02) | | | <0.001 | -167.49 (-209.24, -125.7) | | <0.001 |
|  | Mazandaran | 32.90 (30.43, 35.38) | | | <0.001 | -4.27 (-5.03, -3.52) | | | <0.001 | -9.52 (-10.55, -8.48) | | | <0.001 | -456.45 (-506.72, -406.1) | | <0.001 |
|  | Zahedan | 18.00 (15.83, 20.17) | | | <0.001 | 5.19 (4.53, 5.85) | | | <0.001 | -10.57 (-11.47, -9.66) | | | <0.001 | 132.80 (88.53, 177.06) | | <0.001 |
|  | Yazd | 37.77 (34.73, 40.82) | | | <0.001 | -4.06 (-4.99, -3.13) | | | <0.001 | -13.94 (-15.21, -12.66) | | | <0.001 | -345.27 (-407.35, -283.2) | | <0.001 |
|  | Rafsanjan | 18.18 (15.84, 20.53) | | | <0.001 | -1.12 (-1.84, 0-.41) | | | 0.002 | -7.34 (-8.32, -6.36) | | | <0.001 | -560.74 (-608.30, -513.1) | | <0.001 |
|  | Hoveizeh | 62.25 (60.21, 64.29) | | | <0.001 | -4.59 (-5.21, -3.97) | | | <0.001 | -25.65 (-26.50, -24.79) | | | <0.001 | 363.79 (322.30, 405.27) | | <0.001 |
|  | Shahrekord | 13.09 (10.85, 15.33) | | | <0.001 | 2.08 (1.40, 2.77) | | | <0.001 | -6.54 (-7.48, -5.60) | | | <0.001 | -11.97 (-57.70, 33.74) | | 0.608 |
|  | Bandare Kong | 10.20 (7.92, 12.48) | | | <0.001 | 1.55 (.86, 2.25) | | | <0.001 | -2.91 (-3.86, -1.95) | | | <0.001 | -173.22 (-219.74, -126.7) | | <0.001 |
|  | Urmia | 9.87 (7.55, 12.19) | | | <0.001 | -0.42 (-1.12, 0.28) | | | 0.245 | -2.78 (-3.75, -1.81) | | | <0.001 | -466.87 (-513.97, -419.7) | | <0.001 |
|  | Ardabil | 18.53 (16.46, 20.61) | | | <0.001 | -0.09 (-0.72, 0.53) | | | 0.765 | -7.78 (-8.65, -6.92) | | | <0.001 | -13.23 (-55.53, 29.06) | | 0.540 |
|  | Sabzevar | 31.79 (29.20, 34.38) | | | <0.001 | -6.39 (-7.18, -5.60) | | | <0.001 | -10.46 (-11.54, -9.37) | | | <0.001 | -191.21 (-244.03, -138.4) | | <0.001 |
|  | Dena | 23.69 (21.13, 26.25) | | | <0.001 | -5.86 (-6.64, -5.08) | | | <0.001 | -6.06 (-7.13, -4.99) | | | <0.001 | 2.07 (-50.16, 54.31) | | 0.938 |
|  | Kavar | 30.67 (28.38, 32.95) | | | <0.001 | -2.28 (-2.97, -1.58) | | | <0.001 | -11.21 (-12.16, -10.25) | | | <0.001 | -525.30 (-571.63, -478.9) | | <0.000 |

^†^Models were adjusted for age, time, sex, marital status, education, socioeconomic status (SES), body mass index (BMI), smoking, physical Activity, DMF index, sleep duration, hypertension, diabetes, Cardiovascular Disease, Metabolic Syndrome and interview center. total energy intake was adjusted for macronutrients -^††^_Coefficient (95% Confidence Interval)_
